# Natural Deep Eutectic Solvents for the Extraction of Phenyletanes and Phenylpropanoids of *Rhodiola rosea* L.

**DOI:** 10.3390/molecules25081826

**Published:** 2020-04-16

**Authors:** Alexander N. Shikov, Vera M. Kosman, Elena V. Flissyuk, Irina E. Smekhova, Abdelhameed Elameen, Olga N. Pozharitskaya

**Affiliations:** 1 St. Petersburg State Chemical Pharmaceutical University, Prof. Popov, 14, 197376 Saint-Petersburg, Russia; elena.flisyuk@pharminnotech.com (E.V.F.); irina.smekhova@pharminnotech.com (I.E.S.); 2 St. Petersburg Institute of Pharmacy, Leningrad Region, Vsevolozhsky District, Kuzmolovo P 245, 188663 Saint-Petersburg, Russia; kosmanvm@mail.ru; 3 Norwegian Institute of Bioeconomy Research, Pb 115, NO-1431 Ås, Norway; 4 Murmansk Marine Biological Institute of the Kola Science Center of the Russian Academy of Sciences (MMBI KSC RAS), Vladimirskaya, 17, 183010 Murmansk, Russia; olgapozhar@mail.ru

**Keywords:** *Rhodiola rosea*, extraction, HPLC, optimization, salidroside, tyrosol, rosavin, rosin, cinnamyl alcohol

## Abstract

The extraction of *Rhodiola rosea* rhizomes using natural deep eutectic solvent (NADES) consisting of lactic acid, glucose, fructose, and water was investigated. A two-level Plackett–Burman design with five variables, followed by the steepest ascent method, was undertaken to determine the optimal extraction conditions. Among the five parameters tested, particle size, extraction modulus, and water content were found to have the highest impact on the extrability of phenyletanes and phenylpropanoids. The concentration of active compounds was analyzed by HPLC. The predicted results showed that the extraction yield of the total phenyletanes and phenylpropanoids (25.62 mg/g) could be obtained under the following conditions: extraction time of 154 min, extraction temperature of 22 °C, extraction modulus of 40, molar water content of 5:1:11 (*L*-lactic acid:fructose:water, mol/mol), and a particle size of rhizomes of 0.5–1 mm. These predicted values were further verified by validation experiments in predicted conditions. The experimental yields of salidroside, tyrosol, rosavin, rosin, cinnamyl alcohol and total markers (sum of phenyletanes and phenylpropanoids in mg/g) were 11.90 ± 0.02, 0.36 ± 0.02, 12.23 ± 0.21, 1.41 ± 0.01, 0.20 ± 0.01, and 26.10 ± 0.27 mg/g, respectively, which corresponded well with the predicted values from the models.

## 1. Introduction

*Rhodiola rosea* L. (accepted name according to the www.theplantlist.org database is *Sedum roseum* (L.) Scop.), known in Russia as the golden root (‘зoлoтoй кoрень’ = ‘zolotoy koren’), is a perennial plant from the Crassulaceae family. *R. rosea* has a long history of traditional use. Dioscorides in 77 CEs have mentioned this plant as Rodia riza in De Materia Medica [1]. The plant is used in Russia, Scandinavian countries, Western Europe, and Asia in traditional medicine and as a food supplement [2,3,4,5,6]. The Soviet Union was the first country in which *R. rosea* was implemented in officinal medicine in 1969 [7]. It is now approved by the European Medicines Agency as a traditional herbal medicine product [8]. There are several clinical studies that confirmed the use of *R. rosea* as an evidence-based adaptogen [2,6,7,9,10,11]. This plant is one of the most important adaptogens. The number of scientific publications about this plant has been growing recently [12].

**Figure 1:**
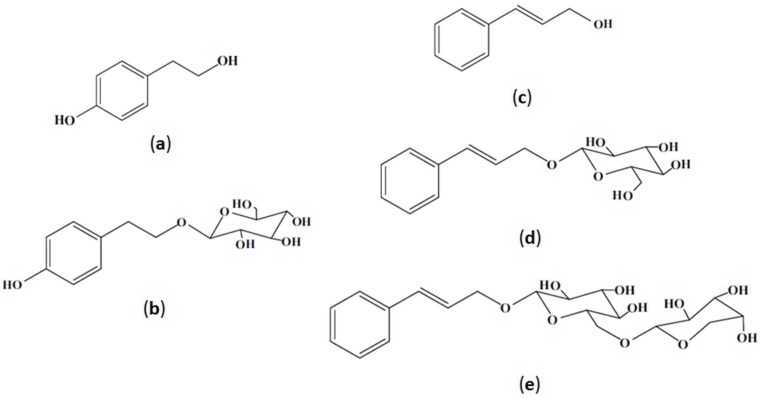
The most important markers of *R. rosea*: (**a**) tyrosol; (**b**) salidroside; (**c**) cinnamyl alcohol; (**d**) rosin; and (**e**) rosavin.

More than 150 biologically active compounds have been identified in *R. rosea* [6,8,13,14,15]. They belong to derivatives of alkanols, benzyl and phenols, phenylethanes, gallic acid, phenylpropanoids, flavonoids, monoterpenoids, triterpenes, and others [13,15,16,17,18,19,20]. Among the other phenyletanes is tyrosol (**a**) and its derivative salidroside (**b**), phenylpropanoids: cinnamyl alcohol (**c**), its glycosides rosin (**d**), and rosavin (**e**), which are believed to be the most important markers of *R. rosea* and are responsible for its pharmacological activity (Figure 1) [2,10,11,19,21]. 

Rhizomes of *R. rosea* are used in traditional and officinal medicine in the form of aqueous infusion/decoctions or as ethanol-based tincture/extract [2,3,5,7]. Supercritical carbon dioxide fluid extraction [22], microwave-assisted, as well as ultrasonic-assisted extractions (UAE) with ion liquids [23,24] were proposed as alternatives for the isolation of active principles of *R. rosea*. A number of imidazolium-based ionic liquids (ILs) with inorganic anions were proposed for the extraction of salidroside and tyrosol from *R. rosea* [25]. ILs were composed of organic cations and inorganic anions. Recently, Tsvetov et al. demonstrated the isolation of cinnamyl alcohol and salidroside from *R. rosea* using deep eutectic solvents (DES) [26,27]. DESs are considered to be green solvents and represent eutectic mixtures of a hydrogen bond donor and a hydrogen bond acceptor. The application of these solvents for the extraction of biologically active compounds from natural sources was extended intensively [28,29,30,31]. When a DES was prepared by the combination of a hydrogen bond donor and an acceptor of natural origin, it was called a natural deep eutectic solvent (NADES).

The history of NADES began in 2011, when a group of scientists, led by Prof. R. Verpoorte from Leiden University [32], hypothesized that there was a third liquid phase in all living organisms. This phase showed phenomenal solubility in various non water-soluble small molecules and biopolymers. The authors proposed to use a NADES term for eutectic mixtures of two or more natural compounds, among choline chloride, citric acid, malic acid, maleic acid, glucose, fructose, sucrose, water, etc. The interest in NADES is growing gradually. NADESs have been reported to be suitable for the extraction of anthocyanins [33,34,35], anthraquinones [36], catehins [37], flavonoids [38,39,40], phlorotannins [41], polysaccharides [42], etc.

NADESs are environmentally friendly, inexpensive, could be easily prepared, have low toxicity, and are pharmaceutically acceptable [31,43,44]. NADES has been suggested as a promising replacement for conventional organic solvents for the extraction of natural products [36,40]. Ultrasound-assisted extraction (UAE) was considered to be a suitable technique for extraction with NADES. UAE can accelerate the mass transfer by disrupting plant cell walls [45].

The aim of this study was to develop an effective NADES-UAE method for the extraction of phenyletanes and phenylpropanoids from *R. rosea* as a possible replacement for conventional 40% ethanol. In the present study, a Plackett–Burman design [46] has been employed to determine the significant factors that affect the extraction of phenyletanes and phenylpropanoids from *R. rosea* using NADES. The results of the optimization of extraction by the steepest ascent method was that an excellent extraction yield for cinnamyl alcohol, rosavin, rosin, salidroside, and tyrosol was achieved. As far as we known, this study is the first to use NADES for the extraction of these markers from *R. rosea*.

## 2. Results and Discussion

### 2.1. Extractability of Phenyletanes and Phenylpropanoids from R. rosea by NADES and 40% Ethanol

The selection of a suitable solvent for extraction is an important step in the isolation of active compounds from medicinal plants and largely defines the yield and composition of the herbal medicine products. Petrochemical solvents used in routine extraction have a lot of disadvantages. The replacement of petrochemical solvents with green solvents is challenging. In order to be green and ecologically friendly, NADES is often implemented for the extraction of polyphenolic compounds from medicinal plants [33,34,35]. Extracts of *R. rosea* have been extensively used in pharmaceuticals, food, and the cosmetic industry. To evaluate NADES as a possible replacement, in this study, its extraction efficacy was compared to the efficacy of a conventional solvent (40% aqueous ethanol), which is used for the preparation of officinal *R. rosea* extract [7].

Recently, DES, containing a mixture of choline chloride, malonic acid, and methanol, was suggested as an effective solvent for the recovery of salidroside from *R. rosea* [27], while DES containing choline chloride, glycerin, and water was considered to be optimal for the extraction of cinnamyl alcohol from *R. rosea* [26]. To the best of our knowledge, this is the first study to investigate the application of NADES for the extraction of the five key markers (rosavin, salidroside, rosin, cinnamyl alcohol, and tyrosol) from *R. rosea*.

Lactic acid–glucose NADES was used for the extraction of anthocyanins [34] and phenolics from some folk plants [47]. Lactic acid-based NADES has been used for the extraction of polyphenols from Greek medicinal plants [48]. Based on these literature data, we tested a number of NADES comprising *L*-lactic acid, glucose, and fructose in different rations for the extraction of phenyletanes and phenylpropanoids from *R. rosea*. The most interesting were NADES LG (lactic acid:glucose:H_2_O (6:1:6)); NADES LF1 (lactic acid:fructose:H_2_O (5:1:1)), and NADES LF2 (lactic acid:fructose:H_2_O (5:1:5)). The high-performance liquid chromatography (HPLC) method was applied for the analysis of cinnamyl alcohol, rosavin, rosin, salidroside, and tyrosol in *R. rosea* extracts.

The efficacy of the most interesting NADES and conventional aqueous alcohol for the recovery of the main active compounds from *R. rosea* by UAE is shown in Table 1. The NADES with fructose were more potent for the extraction of phenyletanes and phenylpropanoids from *R. rosea*. Increasing the polarity of NADES by adding water led to an increase in the yield of cinnamyl alcohol, rosavin, rosin, and salidroside, while the yield of tyrosol was decreased. The application of LF2 was more favorable for the replacement of ethanol. Therefore, LF2 solvent was considered to be the most promising and was selected for further experiments on the optimization of extraction conditions.

### 2.2. Optimization of Extraction Conditions

Following previous studies [35,49,50], particle size, extraction time, temperature, extraction modulus (solid-to-solvent ratio), and molar water content were studied as key parameters affecting the efficacy of the extraction of salidroside, tyrosol, rosavin, rosin, and cinnamyl alcohol (Table 2).

The Plackett–Burman design, which is an efficient way to identify the important factors among a large number of variables [51], was used in the present study to screen the important variables that significantly influenced phenyletanes and phenylpropanoids extraction from *R. rosea* using NADES. The design of the experiment was carried out using the Plackett–Burman central composite design of five variables at two levels (Table 3).

The mathematical regression models for the yield of salidroside (Y1), tyrosol (Y2), rosavin (Y3), rosin (Y4), cinnamyl alcohol (Y5), and sum of all markers (Y5) are shown below in the form of coded levels:

Y1 = 5.29 − 0.92X1 + 0.31X2 − 0.073X3 + 1.24X4 + 1.14X5,(1)

Y2 = 0.18 − 0.023X1 + 0.029X2 + 0.012X3 + 0.054X4 + 0.006X5,(2)

Y3 = 6.06 − 1.48X1 + 0.71X2 + 0.20X3 + 1.05X4 + 1.13X5,(3)

Y4 = 0.520 − 0.120X1 + 0.035X2 + 0.017X3 + 0.084X4 + 0.140X5,(4)

Y5 = 0.037 − 0.010X1 − 0.0014X2 − 0.0088X3 − 0.013X4 + 0.019X5,(5)

Y6 = 12.090 − 2.574X1 + 1.094X2 + 0.146X3 + 2.411X4 + 2.438X5,(6)

where X1, X2, X3, X4, and X5 are the particle size, extraction time, extraction temperature, extraction modulus (solid-to-solvent ratio), and molar water content in NADES, respectively. ANOVA was performed to evaluate the optimal conditions of the NADES-UAE and the relationship between the response and the variables. Table 4 shows the ANOVA results for the models (Equations (1)–(6)).

A model F-ratio of more than 37.29 (F_table_ = 2.13 < F_model_) for all the models was significant. Since the *p*-values for all the models were less than 0.05, there is a statistically significant relationship between the variables at the 95.0% confidence level. The regression analysis of the data revealed that the coefficients of determination (R^2^) for all the models were more than 94.9, suggesting that these models could be used to describe the extraction process.

Among the five parameters, particle size (X1) and extraction modulus (X4) were found to have the highest impact on the phenyletanes and phenylpropanoids yield, as given by the highest linear coefficients (Equations (1)–(6)). Our results showed a clear inverse correlation of the extraction yield with particle size due to the increase of interphase contact surface, permeability of solvent into material, and facilitation of mass exchange [49,52,53]. Molar water content (X5) has positive linear effects for all the markers tested. The addition of water to the NADES is necessary to facilitate the extraction efficacy of biomolecules due to the reduction of the viscosity of solvents (therefore amounting to better mass-transfer rates) and increasing of the polarity [34,40,43]. However, the increase of water content became less significant for the extraction of tyrosol and cinnamyl alcohol (Equations (2) and (5)), possibly because of the weakening of the interactions between the NADES and relatively low polarity of aglycons compared to salidroside, rosavin, and rosin representing glycosides (Figure 1). To prevent the possible degradation of active compounds, we prefer to keep the extraction temperature (X3) at a low level.

As shown in Table 3, the efficacy of NADES extraction was not equal to the efficacy of the extraction by 40% ethanol. In order to improve this, extraction conditions (particle size, extraction time, temperature, extraction modulus (solid-to-solvent ratio), molar water content) (Table 2) were further optimized using the steepest ascent method. The path of steepest ascent was based on the zero level of the Plackett–Burman design and moved along the direction in which the extraction yields of salidroside, tyrosol, rosavin, rosin, cinnamyl alcohol, and the sum of the markers increased. The optimal conditions for the simultaneous extraction of the sum of markers, calculated according to our model, were as follows: extraction time 154 min, extraction temperature 22 °C and extraction modulus 1:40, molar water content 5:1:11, particle size of pulverized rhizomes of *R. rosea* 0.5–1 mm, the predicted extraction yield of the total phenyletanes and phenylpropanoids was 25.62 mg/g.

### 2.3. Verification of the Models

Verification experiments for the confirmation of the suitability of the predicted response values were performed under the optimized conditions in three replicates.

The observed extraction yields of salidroside, tyrosol, rosavin, rosin, cinnamyl alcohol, and total markers (sum of phenyletanes and phenylpropanoids) were 11.90 ± 0.02, 0.36 ± 0.02, 12.23 ± 0.21, 1.41 ± 0.01, 0.20 ± 0.01, and 26.10 ± 0.27 mg/g, respectively, and these were largely consistent with the predicted values. These findings indicated that the established linear models were statistically reliable and reasonable. The extraction yields of the five phenyletanes and phenylpropanoids, and the sum of the target compound from *R. rosea*, were significantly reproducible. The application of NADES LF2 in optimal conditions is a good alternative to EtOH for the extraction of salidroside, rosavin, and rosin, while the recovery of tyrosol and cinnamyl alcohol was lower.

The herbal material has high variability. The place of plant collection, vegetative phase, drying conditions, and other factors affect the content of active compounds. However, we have mentioned that the contents of rosavin and salidroside recovered by NADES were much higher than what was obtained using the other various methods, e.g., 4.11 and 0.93 [54], 3.68 and 1.62 [55], 3.61 and 3.79 [56], 3.50–2.70 [21], 9.77 and 1.81 [57], and 4.20 and 1.20 [58], respectively; all the amounts are given in mg/g. The rosin content in NADES extract was also higher compared to 0.53 mg/g [54], 0.80 mg/g [21]. The maximal content of salidroside after extraction by 1-alkyl-3-methylimidazolium-type ionic liquids was 3.3 mg/g, while the content of tyrosol was 0.75 mg/g [25]. Tsvetov et al. [26,27] have not provided quantitative data for cinnamyl alcohol and salidroside; therefore, we are unable to compare the efficacy of NADES and the DES extraction of *R. rosea*.

## 3. Materials and Methods

### 3.1. Materials and Reagents

The rhizomes of *R. rosea* L. were harvested from a single clone cultivated in the germplasm of NIBIO (Norwegian Institute for Bioeconomy Research, Ås, Norway). This clone was vegetative propagated since 2006. The Norwegian *R. rosea* germplasm collection is known to have high genetic diversity [59] and a high content of bioactive compounds [60]. Voucher specimens SVND is deposited in herbarium of NIBIO, Norway. The rhizomes were dried and pulverized with a disintegrator. The pulverized material was passed through a sieve; a fraction 1–2 mm was taken and stored in a sealed plastic bag before use. Analytical grade chemicals, L-lactic acid, glucose, fructose, and solvents for extraction and HPLC were purchased from local chemical suppliers. Standard reference substance salidroside, tyrosol, rosavin, rosin, and cinnamyl alcohol were purchased from Sigma Chemical Co. (St Louis, MO, USA). The stocks of standard reference solutions were completely dissolved in methanol to provide concentrations that facilitated optimal calibration ranges.

### 3.2. HPLC Analysis

Salidroside, tyrosol, rosavin, rosin, and cinnamyl alcohol were simultaneously quantitated using a Shimadzu LC-20 series liquid chromatography system equipped with two LC-20AD pumps, a DGU-20A3 degasser, an SPD-M20A diode-array detector, and a SIL-20A autoinjector. The reference substances were chromatographically separated on a Luna C18 (2) (4.6 × 250 mm, 5 µm) (Phenomenex, Torrance, CA, USA) column with a 3.0 mm pre-column with the same sorbent (Phenomenex, Torrance, CA, USA). The mobile phase consisted of 0.03% trifluoroacetic acid aqueous solution (A) and acetonitrile (B), and the following gradient elution program was used for separation: 0–30 min, 10–40% B; and 30–35 min, 10% B. The flow rate was 1.0 mL/min, the injection volume was 20 µL, the column temperature was 25 °C, and the detection wavelength was done at 254 nm (rosavin, rosin, cinnamyl alcohol) and 280 nm (salidroside and tyrosol). LCsolution PC software (Shimadzu, Kyoto, Japan) was used for chromatograms registration and development. All of the samples were filtered through a 0.45 µm filter prior to HPLC analysis [54].

The standard curves for salidroside, tyrosol, rosavin, rosin, and cinnamyl alcohol were Y = 0.0002x + 0.1381 (R^2^ = 0.9998, *n* = 10), Y = 0.00009x + 0.1161 (R^2^ = 1.0000, *n* = 10), Y = 0.00002x − 0.1987 (R^2^ = 1.0000, *n* = 10), Y = 0.00002x − 0.2313 (R^2^ = 0.9999, *n* = 10), Y = 0.00001x − 0.755 (R^2^ = 0.9995, *n* = 10), respectively, where X is the peak area of the analyte, and Y is the concentration of analyte (μg/mL). The linearity range for salidroside, tyrosol, rosavin, rosin and cinnamyl alcohol were 0.25–125, 0.25–250, 0.25–250, 0.2–200, and 0.25–250 μg/mL, respectively. The Limit of Quantification (LOQ) for the reference substances were 0.25 μg/mL for salidroside, tyrosol, rosavin, and cinnamyl alcohol, and 0.2 μg/mL for rosin.

### 3.3. NADES Preparation

All NADES were prepared according to the reported method [61]. Lactic acid (L) and hydrogen bond donor (HDB) glucose (G) or fructose (F) at the respective molar ratio were directly weighed, and the mixture was stirred in the sealed flask at 50 °C for 90 min until a homogeneous transparent colourless liquid was formed. NADES solutions with water were prepared from the starting solvent by adding a certain amount of water in the respective weight ratio.

### 3.4. UAE with Different Solvents

The pulverized *R. rosea* L. (1.00 g) was placed in a beaker flask, 20 mL of the different NADESs was added, shaken vigorously manually for a few seconds to form a slurry, and then the flask was placed in a temperature-controlled ultrasonic cleaner (Sapphire UZV-4.0, Moscow, Russia), at a sonication power of 50 W, a frequency of 35 kHz, and extracted for 60 min at 36 °C [62]. Then, 40% (v/v) aqueous ethanol was also used for comparison. After extraction was completed, the solutions were collected and centrifuged at 3000 rpm for 15 min in a table centrifuge (BIOSAN LMC-3000, Riga, Latvia). A supernatant of 5 mL volume was carefully removed, and a dilution of 5× was made. Finally, the diluted solutions were filtered through a 0.45 µm filter and analyzed through HPLC. These experiments were repeated three times.

### 3.5. Experimental Design and Statistical Analysis

In this study, an 8-run Plackett–Burman design was applied to evaluate five factors, including particle size, extraction time, temperature, extraction modulus (solid-to-solvent ratio), and molar water content. Each variable was examined at two levels: −1 for the low level and +1 for the high level (Table 2). All experiments were conducted in duplicate, and the average value of the extracted phenyletanes and phenylpropanoids was used for statistical analysis. Table 3 illustrates the variables and their corresponding levels were used in the experimental design. The values of two levels were set according to the preliminary experimental results (Table 2).

The steepest ascent method is a procedure for moving along the maximum increase in the response [49]. The direction of the steepest ascent is the direction in which the response increases most rapidly by increasing or decreasing the values of the significant factors. The zero level of Plackett–Burman design was identified as the base point of the steepest ascent path. The step along the path was determined by the estimated coefficient from Equation (6) and the practical experience.

### 3.6. Data Analysis

Statgraphics Centurion 16.2 (Statpoint Technologies, Inc., Warrenton, VA, USA) was used to design the experiments, as well as for the regression and graphical analysis of the experimental data obtained.

## 4. Conclusions

In this study, the extraction of phenyletanes and phenylpropanoids from *R. rosea* rhizomes with NADES was investigated. L-lactic acid:fructose-based NADES could be considered a viable alternative to 40% aqueous ethanol for the extraction of salidroside, tyrosol, rosavin, rosin, and cinnamyl alcohol. The Plackett–Burman design, followed by the steepest ascent method, was used for the optimization of extraction conditions. The optimal extraction conditions were as follows: extraction time 154 min, extraction temperature 22 °C and extraction modulus 1:40, molar water content 5:1:11 (L-lactic acid:fructose:water, mol/mol), particle size of pulverized rhizomes 0.5–1 mm. Under this condition, the yields of salidroside, tyrosol, rosavin, rosin, cinnamyl alcohol and total markers (sum of phenyletanes and phenylpropanoids) were 11.90 ± 0.02, 0.36 ± 0.02, 12.23 ± 0.21, 1.41 ± 0.01, 0.20 ± 0.01, and 26.10 ± 0.27 mg/g, respectively, which corresponded well with the predicted values of the models. The application of NADES LF2 in optimal conditions is a good alternative to EtOH for the extraction of salidroside, rosavin, and rosavin, while the recovery of tyrosol and cinnamyl alcohol was lower.

## Figures and Tables

**Table 1:** Extraction yields of phenyletanes and phenylpropanoids from *R. rosea* by ultrasonic-assisted extractions (UAE) with natural deep eutectic solvent (NADES) and 40% aqueous ethanol.

Solvent	Extraction Yield (Mean ± SD), mg/g				
	Cinnamyl Alcohol	Rosavin	Rosin	Salidroside	Tyrosol
NADES LG	0.11 ± 0.01	6.28 ± 0.02	0.32 ± 0.01	4.07 ± 0.02	0.33 ± 0.06
NADES LF1	0.14 ± 0.02	8.42 ± 0.48	0.42 ± 0.01	5.66 ± 0.01	0.61 ± 0.02
NADES LF2	0.26 ± 0.03	10.38 ± 0.03	1.11 ± 0.02	7.01 ± 0.06	0.33 ± 0.01
EtOH 40%	0.50 ± 0.01	12.11 ± 0.05	1.08 ± 0.02	10.70 ± 0.23	1.25 ± 0.01

**Table 2:** The main factors and their variation levels for extracting phenyletanes and phenylpropanoids from *R. rosea* by NADES-UAE.

Levels	Factors				
	X1 (Particle Size, mm)	X2 (Extraction Time, min)	X3 (Temperature, °C)	X4 (Extraction Modulus)	X5 (Molar Water Content)
Main level (0)	0.5 … 2.0	60	36	1:30	5:1:5
High level (+1)	2.0 … 3.0	90	50	1:40	5:1:7
Low level (−1)	0 … 0.5	30	22	1:20	5:1:3

**Table 3:** Plackett–Burman experiment design with the independent variables.

Run	Factors					Extraction Yield (Y_i_, mg/g) *					
	X1	X2	X3	X4	X5	Salidroside (Y1)	Tyrosol (Y2)	Rosavin (Y3)	Rosin (Y4)	Cinnamyl Alcohol (Y5)	Sum (Y6)
1	+1	+1	+1	+1	+1	7.40 ± 0.06	0.25 ± 0,03	8.22 ± 0.07	0.72 ± 0.01	0.029 ± 0.002	16.62 ± 0.13
2	−1	−1	+1	+1	+1	7.80 ± 0.14	0.25 ± 0.01	8.65 ± 0.30	0.81 ± 0.01	0.040 ± 0.000	17.55 ± 0.45
3	−1	+1	+1	−1	−1	3.64 ± 0.32	0.17 ± 0.02	5.81 ± 0.12	0.44 ± 0.01	0.026 ± 0.001	10.07 ± 0.20
4	+1	−1	+1	−1	−1	2.04 ± 0.06	0.10 ± 0.01	2.37 ± 0.12	0.19 ± 0.01	0.019 ± 0.001	4.71 ± 0.07
5	+1	+1	−1	−1	+1	4.23 ± 0.01	0.13 ± 0.00	4.63 ± 0.01	0.43 ± 0.01	0.061 ± 0.001	9.47 ± 0.03
6	−1	−1	−1	−1	+1	6.31 ± 0.00	0.11 ± 0.00	7.26 ± 0.01	0.70 ± 0.00	0.095 ± 0.007	14.47 ± 0.03
7	−1	+1	−1	+1	−1	7.17 ± 0.08	0.29 ± 0.01	8.46 ± 0.01	0.64 ± 0.02	0.028 ± 0.004	16.57 ± 0.11
8	+1	−1	−1	+1	−1	3.75 ± 0.07	0.15 ± 0.02	3.11 ± 0.14	0.26 ± 0.01	0.00 ± 0.00	7.26 ± 0.21

X1: particle size; X2: extraction time; X3: extraction temperature; X4: extraction modulus; X5: molar water content; * Mean ± SD.

**Table 4:** ANOVA of the linear models (1)–(6).

Model	F-Ratio	*p*-Value	R^2^
Y1, salidroside	38.01	0.0000	95.00
Y2, tyrosol	45.09	0.0000	95.75
Y3, rosavin	37.29	0.0000	94.91
Y4, rosin	47.92	0.0000	95.99
Y5, cinnamyl alcohol	42.47	0.0000	95.50
Y6, sum of markers	38.11	0.0000	95.01
